# What did the Go4Health policy research project contribute to the policy discourse on the sustainable development goals? A reflexive review

**DOI:** 10.1186/s12992-018-0367-4

**Published:** 2018-05-16

**Authors:** Vannarath Te, Nadia Floden, Sameera Hussain, Claire E. Brolan, Peter S. Hill

**Affiliations:** 1 School of Public Health, The University of Queensland, Herston Road, Brisbane, Australia; 2 National Institute of Public Health, Ministry of Health, Phnom Penh, Cambodia; 3 Canadian Society for International Health, Ottawa, Canada; 4 School of Epidemiology and Public Health, University of Ottawa, Ottawa, Canada; 5 Dalla Lana School of Public Health, University of Toronto, Toronto, Canada

**Keywords:** Sustainable development goals, Right to health, Universal health coverage, Marginalized communities, Global health governance, Integration, Policy

## Abstract

**Background:**

In 2012, the European Commission funded Go4Health—Goals and Governance for Global Health, a consortium of 13 academic research and human rights institutions from both Global North and South—to track the evolution of the Sustainable Development Goals (SDGs), and provide ongoing policy advice. This paper reviews the research outputs published between 2012 and 2016, analyzing the thematic content of the publications, and the influence on global health and development discourse through citation metrics.

**Findings and discussion:**

Analysis of the 54 published papers showed 6 dominant themes related to the SDGs: the formulation process for the SDG health goal; the right to health; Universal Health Coverage; voices of marginalized peoples; global health governance; and the integration of health across the other SDGs. The papers combined advocacy---particularly for the right to health and its potential embodiment in Universal Health Coverage—with qualitative research and analysis of policy and stakeholders. Go4Health’s publications on the right to health, global health governance and the voices of marginalized peoples in relation to the SDGs represented a substantial proportion of papers published for these topics. Go4Health analysis of the right to health clarified its elements and their application to Universal Health Coverage, global health governance, financing the SDGs and access to medicines. Qualitative research identified correspondence between perceptions of marginalized peoples and right to health principles, and reluctance among multilateral organizations to explicitly represent the right to health in the goals, despite their acknowledgement of their importance. Citation metrics analysis confirmed an average of 5.5 citations per paper, with a field-weighted citation impact of 2.24 for the 43 peer reviewed publications. Citations in the academic literature and UN policy documents confirmed the impact of Go4Health on the global discourse around the SDGs, but within the Go4Health consortium there was also evidence of two epistemological frames of analysis—normative legal analysis and empirical research—that created productive synergies in unpacking the health SDG and the right to health.

**Conclusion:**

The analysis offers clear evidence for the contribution of funded programmatic research—such as the Go4Health project—to the global health discourse.

## Background

Research on the development of health policy—particularly policy impacting Low- and Middle-Income Countries—has been quite limited [1] despite evidence of increased funding for health systems and policy research [2]. While attention is usually given to health policy as a product or output, the process of policy making—how policies come to attention, how they are formulated and implemented, and what forces influence the decision-making process—is equally important and worth analyzing [3, 4]. The analysis of the policy process contributes to the knowledge on the development of policy agendas and priorities, and the influence of stakeholders in the policy context [5]. Buse [6] advocates for prospective policy analysis—“analysis which seeks to understand the unfolding political-economy environment of policy change so as to support stakeholders to more effectively engage in policy processes”—which is then capable of supporting and shaping health policy making. This policy analysis is commonly undertaken by advocacy coalitions, highly-integrated policy networks [6], with the potential to set agendas and reach decision makers [7].

Go4Health is an example of an academic research coalition that strongly advocated the right to health as a central component in the post-2015 international health agenda, as debate focused on the development agenda that would succeed the Millennium Development Goals (MDGs) [8]. Launched in 2012, ‘Go4Health’—standing for goals and governance for global health—was co-funded by the European Commission’s Seventh Framework Programme and Australia’s National Health and Medical Research Council to analyze the development of the Sustainable Development Goals (SDGs), providing advice to the European Commission and advocating positions in the global policy discourse [9]. The consortium included 13 research partners: ten academic institutions—including academics in global public health, international law and human rights [8], and four health rights advocacy organizations, from six continents combining the Global North and the Global South (Africa, Asia, Australia, Europe, North and Latin America) (Table 1).

**Table 1:** Go4Health research consortium

Institution	Country	Type of Institution
Institute of Tropical Medicine (ITM)	Belgium	Research and training institute
University of Heidelberg, Institute of Public Health (Universitätsklinikum Heidelberg)	Germany	University
The O’Neill Institute for National and Global Health Law, Georgetown University	United States of America	University
University of Oxford/ University of Edinburgh,	United Kingdom	University
Medico International, Berlin	Germany	Human rights advocacy organization
Center for Health, Human Rights and Development	Uganda	Human rights advocacy organization
University of Nairobi	Kenya	University
James P. Grant School of Public Health, BRAC University	Bangladesh	University
Centro de Estudios para la Equidad y Gobernanza en los Sistemas de Salud (Center for the Study of Equity and Governance in Health Systems)	Guatemala	Research and human rights advocacy organization
London School of Hygiene and Tropical Medicine	United Kingdom	University
The University of Queensland	Australia	University
Dalla Lana School of Public Health, University of Toronto	Canada	University
Norwegian Human Rights Center, University of Oslo	Norway	University

The 35 researchers listed in the original proposal represented a rich and diverse complex of actors. Research disciplines included public health, health systems and policy research, international law (including taxation), human rights and international relations. All advocacy organizations had a history of research engagement. Institutions worked as teams linked to joint work packages; team members ranged from lead academics extensively published in their fields [8] to emerging academics—four doctorates were successfully defended from the research. The initial research teams included 11 females, increasing by five over the life of the project; cultural diversity was rich.

The objectives set for the research were to examine the context of the post-2015 global development agenda and its roots in the MDGs; the perceptions of marginalized communities on global health and development priorities; the roles of multilateral agencies in the development of the SDGs; and finally, their implications for states and global health governance [9, 10]. Funding from the research ensured participation in many of the seminars and conferences associated with the development of the SDGs, and regular consultation with European and multilateral representatives responsible for their agencies’ engagement in the SDGs. Early in the research, Go4Health advocated that the post-2015 health development goals should incorporate Universal Health Coverage (UHC) anchored in the right to health [10]. The overarching health goal proposed by the Go4Health was “the realization of the right to health for everyone” with the twin targets of UHC anchored in the right to health and the creation of a healthy social, economic and natural environment for all [10]. This early commitment by Go4Health to a proposed goal that embraced UHC marked a decision to combine its roles in policy research with that of advocacy—particularly around the right to health. This dual role of Go4Health is implicit in the structure of the consortium. The diversity in Go4Health’s research teams also introduced differences in perceptions of research positioning and in analytic approaches. This divergence—and its synthesis—becomes more evident in the content analysis of its research output. Debate within Go4Health team meetings was, in part, pragmatic—choices of journals for publication, and the audiences targeted, tensions between the delays of peer review and the authority it confers, the accessibility of open access publication compared to its costs. At times, it was ideological and theoretical: the balance between advocacy and research (and what was appropriate research), the desire for participatory approaches in a project that did not resource them, the boundaries of argument and imputation, and of research and policy.

In health systems and policy research there is ample research *for* policy but limited research *on* policy—the policy process and policy implementation [1]. Ghaffar et al. [1] argue that the lack of research on policy analysis is a critical gap in the field, and urge more investment by the global health community to support health policy analysis. Go4Health provides an exemplar of what is possible when peak research funding agencies support a contemporaneous program of policy research of global significance, with the potential to shape the global discourse around the SDGs. The completion of the research has provided the basis for a reflexive analysis of the themes that Go4Health addressed: this paper explores both the evolution and synthesis of the content of the Go4Health research, and through citation metric analysis, some quantification of their publications.

## Methods

### Thematic analysis

In order to understand the contribution of Go4Health to the post-2015 discourse, we reviewed all research products listed on the Go4Health project website (54 publications), between November 2012 to December 2016. As a result, the analysis focuses on publications released (and cited) during the period of the development of the SDGs and into their first year of implementation [11]. Qualitative thematic analysis was employed to explore the thematic content of the whole corpus of Go4Health research product. The analysis aims to identify, analyze, and report common patterns or themes within the data, seeking to identify the essential narrative of Go4Health’s publications [12]. To formulate the key themes, the first three authors (VT, NF, and SH) analyzed the Go4Health research proposal to establish a priori themes, then reviewed the abstracts of all the publications, using open coding to elaborate emergent themes and codes [13]. The themes and codes were corroborated in discussions with the supervising authors (CEB and PSH). SH, CEB and PSH were researchers within the Go4Health team. The agreed themes resulting from the analysis were:

- Formulation process for the SDG health goal (SDG3)
- Right to health
- Universal health coverage
- Voices of marginalised peoples
- Global health governance
- Integration of health across other SDGs

Based on this agreed framework, full-text review of each publication was then completed for analysis by VT, NF, and SH, using NVivo 11, qualitative research analysis software [14], to code, query, manage, and visualize the data for analysis.

### Citation metrics analysis

In January and February 2017, a rigorous search was conducted using six databases selected for their content coverage and areas of focus: Scopus, Web of Science, EMBASE, Global Health Library, PubMed, Worldwide Political Science Abstracts in ProQuest [15]. The databases reflect the intersectional and interdisciplinary nature of issues pertaining to the SDGs, global health and development more broadly.

The citation searches used keywords based on the main themes identified in the thematic analysis of the Go4Health project publications as an initial search, with keywords then combined and expanded, based on the six themes (Table 2).

**Table 2:** Keyword combinations for framing search and searches on identified themes

Framing Search	Keywords
	[((post-2015 goal*) OR (post-2015 development agenda) OR (SDG*) OR (health goal*)) AND keywords in each theme]
Thematic Searches	Keyword Combinations
1. Formulation process for the SDG health goal (SDG3)	AND ((formulat* OR develop*) AND ((SDG health goal) OR (SDG3)))
2. Right to health	AND (“right to health” OR “health rights”)
3. Universal health coverage	AND (“universal health coverage” OR “universal health care”)
4. Global health governance	AND (“global health governance” OR “global governance for health” OR “governance for global health”)
5. Voices of marginalized peoples	AND (marginali?ed. people* OR marginali?ed. population* OR “community participation” OR “participatory decision making”)
6. Integration of health across other SDGs	AND ((health integrated agenda) OR “health determinants” OR “determinants of health”)

Only publications written in English and published between 2012 and 2016 were included in the search. The analysis of the selected databases identified the patterns of citation within each data base, and enabled an analysis of the citation metrics of the Go4Health publications. This was undertaken in February 2017, following the principles outlined in the 2015 Leiden Manifesto [16], using SciVal [17] and the Scopus database, with 32 Go4Health publications available for analysis. SciVal is an analytical tool equipped with the function to assess research performance of 8500 research institutions worldwide. It offers capabilities to conduct bibliometric measures using data from Scopus, analyze research trends, provide benchmark in comparison to peers, and visualize research performance of an institution, a group of researchers, and individual researchers [17, 18]. Field-weighted citation impact is the ratio of citations received relative to the expected world average for the subject field, publication type and publication year. The field-weighted citation impact of the “World” or entire Scopus database is 1.00; with field-weighted citation impact > 1.00 indicating more frequent citation based on the global average for similar publications, and < 1.00 less than would be expected [18]. To identify Go4Health publications cited in United Nations (UN) documents, the United Nations Official Document System [19] was searched for the most prominent Go4Health publications. The publications selected had each been identified in at least four of the six databases that were searched.

## Findings and discussion

In order to ensure greater clarity in the presentation of the overall narratives of the Go4Health research, the findings of both thematic and relevant citation metrics analysis are presented under the six main themes, together with the discussion. For the same reasons, the referencing in this analysis largely privileges Go4Health publications, demonstrating their combined contribution to the development of the six themes in the post-2015 discourse, with relevant supplementary references as appropriate. The findings of the thematic analysis represent a deconstruction of the research product of Go4Health as a whole, bringing together the discourses presented across this literature.

Of the 54 Go4Health publications (2012–2016), 43 are peer-reviewed research or policy commentaries; seven are reports or chapters in reports; and four are posts in blogs or a newsletter. Citation analysis showed that Go4Health publications on the right to health, global health governance, and the voices of the marginalized, had a coherent message and represented a substantial proportion of all published papers identified for those themes in the policy discourse Table 3. shows Go4Health publications as a percentage of all papers published on the identified themes, based on a Scopus search. Collectively, over the period of development and the first year of implementation of the SDGs, Go4Health had an average of 5.5 citations per paper, with a field-weighted citation impact of 2.24. This compares favourably with SciVal’s benchmarks for the related categories of Health Policy papers (3.9 citations per paper, field-weighted citation impact 1.05), and Public Health, Environmental and Occupational Health papers (4.8 citations per paper, field-weighted citation impact 1.0) [17]. Six Go4Health papers were in the top 10% most cited publications globally, based on field-weighted citation impact, and 19 (63.3%) of the publications were published in journals ranked in the top quartile for subject category [17]. Two papers were in the top 1% most cited papers [20, 21], but the most cited paper shared authorship with the Harvard-LSHTM Independent Panel on the Global Response to Ebola [20]. Six papers were cited in UN documentation. In terms of media recognition beyond peer review, seven papers were in the top 5% of all articles scored by Altmetric.

**Table 3:** Go4Health publications compared to all papers published by search theme (source: Scopus, 2012–6)

Theme	Total Publications	Go4Health Publications
1. Formulation process for the SDG health goal (SDG3)	117	3 (3%)
2. Right to health	71	13 (18%)
3. Universal health coverage	191	7 (4%)
4. Global health governance	34	7 (21%)
5. Voices of marginalized peoples	66	6 (9%)
6. Integration of health across other SDGs	334	6 (1.8%)

### Formulation process for the SDG health goal (SDG3)

Given the terms of the European Commission FP7 call, the formulation process for the SDG health goal was a predictable central theme of the Go4Health research. The analysis of the Go4Health papers pointed to both advocacy for the realization of the right to health as a potential post-2015 goal [8, 22]—expressed in the implementation of UHC, and the creation of a healthy global environment—and tracked Go4Health’s positioning in the contemporaneous health goal debates. UHC had been advocated by the World Health Organization (WHO) as the overarching health goal; this was clearly reflected in the 2010 *World Health Report: Health systems financing: the path to universal coverage* [23], and the 2012 WHO *Discussion Paper on health in the post-2015 agenda* [24], defining UHC as “all people receiving the quality health services they need, without being exposed to financial hardship” [25]. Go4Health broadly endorsed WHO’s preceptions of the potential for UHC to address the unfinished tasks of the MDGs, extending that role to engage the global burden of non-communicable diseases (NCDs), health inequity, health security, health systems strengthening, the determinants of health, health as a human right, and health in all policies [22]. There was some early equivocation within Go4Health around the slipperiness of UHC’s definition [26], and subsequent tension around potential dominance of UHC by clinical agendas and the failure to embrace public health strategies [27].

But Go4Health’s core position aligned with WHO’s argument that with UHC as an umbrella health goal—anchored in the right to health—the gains of the MDGs could be consolidated and all these diverse health concerns accommodated [22]. This was also overt in WHO’s formulation of UHC: “UHC is thus a critical component of sustainable development and poverty reduction” and “a practical expression of the concern for health equity and the right to health” [24].

Go4Health pointed to the inclusion of UHC in the 2012 *United Nations Conference on Sustainable Development (Rio + 20)* as a catalyst for strenthening health systems to attain equitable universal coverage [10, 28]. In December 2012, global commitment on UHC was consolidated as the UN General Assembly unequivocally endorsed a Foreign Policy and Global Health resolution on achieving UHC as a “global goal” [29]. This was reiterated in the Sixty-Sixth World Health Assembly in 2013 as means to maximize “health in all life stages” in conjunction with accelerating health-related MDGs and reducing NCD burden [30].

But UHC as an umbrella goal was not universally supported in the post-2015 development debates, and Go4Health documented its unpredictable trajectory [31, 32]. The Global Thematic Consultation on Health in April 2013 [33] included UHC and access to health care as a mechanism for achieving their proposed overarching health goal ‘Maximizing Healthy Lives’. In May 2013, however, the UN Secretary-General’s High-Level Panel of Eminent Persons on the Post-2015 Development Agenda [34] reframed the overarching health goal to ‘Ensure Healthy Lives’, failing to include UHC in either their proposed goal or targets. This shift away from UHC was maintained, in part, when the Sustainable Development Solutions Network suggested ‘Achieve Health and Well-being at All Ages’ as an overarching health goal in June 2013 [35], but included UHC as a way to ensure the provision of primary health services [10]. Go4Health tracked that diversion from the centrality of UHC with some concern [10, 31, 32], but with responsibility for finalizing the post-2015 goals moving from the UN Secretariat to the Intergovernmental Open Working Group on SDGs [36], Go4Health noted UHC’s eventual reinstatement as a health target in the final negotiations [31]. Despite this apparent resolution, advocacy for UHC as a goal persisted: as late as January 2015, the Prince Mahidol Awards Conference was still proposing a compromise goal ‘Progressively Achieve Universal Health Coverage and Ensure Healthy Lives for All’ [32].

In September 2015 the UN General Assembly unanimously endorsed the 17 SDGs and 169 targets [37], with health explicitly positioned in SDG3—‘Ensure healthy lives and promote well-being for all at all ages’—with 9 targets and 4 means of implementation, including the achievement of UHC as target 3.8.

### Right to health

Based on the analysis of both peer-reviewed and other publications, it was clear that the strength of Go4Health’s endorsement of UHC was predicated on its capacity to embody the right to health [10, 22, 38]. In the six databases studied, Scopus identified more Go4Health papers relating to the right to health theme than for any other theme (Table 3), with its nine articles identified in PubMed representing over half of PubMed publications on the right to health and the SDGs during that period (Table 4). Though ten Go4Health publications on the right to health and the SDGs were identified in Worldwide Political Science Abstracts database, the much larger total pool of publications linking these themes (*n* = 2405) has diluted Go4Health’s presence in the field of political science (Table 4).

**Table 4:** Go4Health publications compared to all publications on the right to health search, by database, 2012–16

Database	Total Publications	Go4Health Publications
WPSA	2405	10 (0.4%)
Scopus	71	13 (18%)
Embase	53	12 (23%)
GHL	36	9 (25%)
WoS	48	12 (25%)
PubMed	17	9 (53%)

*WPSA* Worldwide Political Science Abstracts, *GHL* Global Health Library, *WoS* Web of Science

For Go4Health analysts, there is a constant tension between the implicit and explicit reference to the right to health in the post-2015 debate [22, 38–41]. While rights are referred to in the preamble of the UN motion for the SDGs, Go4Health analysis noted that the right to health does not appear in the goals themselves, although the word right(s) is mentioned two times in the SDG3.b means of implementation [37]. Brolan et al.’s [39–41] analysis of the UN resolution on the SDGs expressed concern on the lack of specific reference to the right to health in that resolution, and saw this as reflecting the erosion of rights as a construction in the post-2015 debate. This analysis of why the right to health was not explicit in the post-2015 policy negotiations is the only Go4Health paper directly cited in a UN General Assembly resolution [39, 42].

Go4Health analyses argued for explicit advocacy for the right to health, given the importance of human rights principles in International Human Rights Law [8, 10, 38, 43–45], and its demand for universal access to health care, not just for segments of the community [10, 45–48]. It noted criticism on the MDG’s selective prioritization of a limited range of health issues, and partial coverage [10], arguing that a post-2015 development health goal anchored in the right to health would reduce the health inequities that had increased despite the advances of the MDGs [43, 49].

Given the strength of human rights and international law expertise within the Go4Health research team, Go4Health publications represent a significant contribution to the post-2015 discourse in examining that discourse through the prism of the right to health. They traced its roots to the International Covenant on Economic, Social, and Cultural Rights (Article 12), with its scope and content clarified by General Comment 14 [8, 10, 38, 43, 50, 51]. They argued that the concept of ‘equity’ has been central to the definition of right to health [10, 38], and noted that the right to health does not only cover health care needs but also healthy social, economic and natural environments—the underlying determinants of health—linking this to the concept of ‘Health in All Policies’ [10].

Go4Health papers clearly articulated the seven principles of the right to health, and their inter-relationships. Several papers highlighted the principle of ‘non-discrimination’ and its implications, leading to the principle of ‘prioritization of marginalized peoples’ and the push for ‘participatory-decision making’ [38, 45, 48, 52]. Civil society and community engagement were shown to be central to the realization of that participation. Forman et al. [45] looked specifically at the influence of the principle of ‘progressive realization’ on the principle of ‘minimum core obligations’. These are determinants of essential health needs, raising questions over what and who would be covered by UHC, at what cost. The right to health approaches this by balancing the ‘cost-effectiveness’ principle [44] against the principle of ‘prioritization of marginalized peoples’, and resolving the obligations raised by implementing the principle of ‘shared responsibilities’ [8, 43, 50]. This normative legal analysis framed Go4Health’s aim in analyzing the post-2015 discourse: “to advance and improve on the concept of a global social contract as first articulated in the Millennium Declaration; proposing goals and a governance structure centred on a framework of shared but differentiated responsibilities” [9].

For successful implementation of the right to health, Go4Health argued that a strong ‘global social contract’ is necessary—despite its status as ‘voluntary cooperation’—defined as a political concept capturing the responsibilities of citizens and governments to achieve the agreed goals [9]. The publications also highlighted an urge to determine ‘extra-territorial obligations’ [45, 53] and to foster health systems strengthening, vital for the implementation of the right to health through UHC [8, 10, 43, 50].

But Go4Health analyses also addressed overt challenges to the right to health, particularly in regard to sexual and reproductive health and rights (SRHR). While SRHR were regarded as part of human rights in the 1994 UN Cairo Conference on Population and Development and the follow-up 1995 Beijing World Conference on Women, they were omitted in the initial MDGs, with a reproductive health target only later being added following persistent advocacy [43]. Go4Health analyses noted that while SRHR were integral to all of the UN consultations for the post-2015 goals, it was clear that they remained a sensitive focus of geopolitics [21, 39, 54]. But the insistence of separating reproductive rights from sexual rights—resulting in two separate SDG targets on sexual and reproductive health--revealed growing anxieties around the use of the post-2015 goals to legitimize Lesbian, Gay, Bisexual, and Transgender (LGBT) rights, reflected in interviews with advisors to multilateral organizations [21, 39, 54]. Go4Health advocates unsuccessfully argued that LGBT rights should also be given priority, if “ensuring UHC that meets people’s demands also entails meeting their diverse but particular needs” is to be realized [10].

Other issues around the right to health in the proposed goals were identified in Go4Health publications: the scope of country obligations had not been clearly specified, particularly in regard to primary health care. The principle of ‘progressive realization’ was also vague, potentially allowing some Member States to exploit the vagueness as an excuse for failing to fulfill the ‘minimum core obligations’. Go4Health research identified concerns around the right to health and its derivation from International Human Rights, with some Member States reluctant to comply with health obligations which may potentially clash with their socio-cultural norms [11, 22, 39, 44, 45, 47, 51]. Lastly, the measurability of achievement of progressive realization was seen to be a challenge for states, especially in demonstrating progress towards transformative goals [55].

### Universal health coverage

The early advocacy for UHC created clear overlap with analyses of its position in the post-2015 goals and the formulation of the health goal itself, but UHC itself was also a specific focus of analysis, with Go4Health tracing its identification from the 2005 World Health Assembly resolution and the 2010 *World Health Report: Health systems financing: the path to universal coverage* [38].

This broad support of UHC by Go4Health is clearly conditional—with Go4Health publications comprehensively unpacking the implications of a right to health based UHC. Conflicts surrounding the definition of UHC were often cited: a lack of agreement on definition [40, 44, 55]; different understandings of what UHC actually means in implementation [26, 46]; the risks of UHC being limited to health care services, rather than extending to the social and environmental determinants of health [51, 52]. Go4Health argued that UHC could function as the embodiment of the right to health in the SDGs [38], but this requires a UHC “grounded in the right to health” [44], incorporating the essential principles of the right to health [10, 45, 55]. UHC arguably needs to meet the principle of ‘minimum core obligations’ [45], though the WHO had defined UHC as a challenge to extend coverage, services and financial protection, rather than as a fixed minimum package, as in the essential services of Primary Health Care [8, 10]. Under the influence of the principle of ‘progressive realization’, UHC is a “work in progress” [38] in which Member States need to mobilize the “maximum of their available resources” [8, 45]. Under the right to health, that national obligation under UHC requires “a straightforward confirmation that international assistance is essential, not optional”, based on the principle of ‘shared responsibilities’ [38, 53], and that a framework convention on global health might provide a mechanism for fixing some of its definitional tensions [26]. According to the principle of ‘non-discrimination’, UHC should be underpinned by the ‘equity’ concept [56], engaging the principles of ‘prioritization of marginalized peoples’, ‘participatory-decision making’, and ‘equity’ would also help ensure the appropriate application of the principle of ‘cost-effectiveness’ in an attempt to secure essential health needs [52]. Go4Health publications addressed this concretely, proposing indicators and frameworks to measure equitable access [55].

For successful implementation of UHC, Go4Health advocated the need for new governance structures that would support health systems strengthening is required so that all areas of health issues, but particularly the challenges of mental health and non-communicable diseases, introduced following the MDGS, can be covered and equitably addressed [10, 26, 57, 58].

### Voice of marginalized peoples

This exploration of the right to health in UHC committed Go4Health to consult directly with marginalized communities, working with them on identifying the elements of the essential health needs that could be used as the basis for the new social contract advocated within the new goals. Go4Health sought to prioritize the voices of those whose health is most at risk, specifically, the most marginalized communities in the global South—those whose voices are typically unheard [22, 59]. This representation was limited by resource and opportunity, including Indigenous and migrant communities in Australia, for example [47, 60], but not the Roma in Europe. Marginalized peoples were recognized to have lived through experiences that contribute to a mistrust of those outside their communities, and in the interest of bringing their voices to the global health discourse, Go4Health made efforts to publish the results of community consultations widely [49, 60–63].

Publications documenting these consultations were unanimous in expressing a holistic and communal understanding of health. But they also acknowledged the need for biomedical and facility-based health services that they viewed as embedded in public health systems [49, 60–63]. The limited availability of water, sanitation, and hygiene were critical issues for the communities. The need for improved sanitation systems (in both rural and urban settings), personal hygiene, and waste management were all matters that study participants linked with the need for access to safe water and their health [52, 62]. Along with food, water, and sanitation, community members cited other needs that are essential for health and wellness, such as employment and income generation, housing, education, and a clean natural environment [10, 52, 60, 62].

In interviews with Go4Health researchers it was clear that the current experience of marginalized peoples did not meet their expectations. They described being treated with discrimination by all levels of staff in public health facilities, and subjected to rude behavior and even ridicule [60–62]. This was exacerbated by long waiting times and resource shortages in personnel, medicines and medical supplies. Faced with this, people resorted to making informal out of pocket payments to ensure better care in public health facilities or sought alternatives in the private sector, with traditional medicine a popular choice among respondents because of its ready availability [60, 62, 63].

Communities across the study placed primary responsibility to the state to ensure their health and well-being. Not only were health systems perceived as unresponsive because of a lack of resources, community members reported that local, regional, and national institutions lacked the political will to improve their lives. This left community members feeling hopeless about bringing about changes to the status quo. Instead, they looked to non-state actors like non-governmental organizations (NGOs) for the provision of public services [10, 60–62]. Respondents placed their trust in NGOs to help them develop the necessary capabilities for bringing their voices into decision-making [10]. They went further, expressing a desire to learn more about their rights and how to make effective demands for justice in the health system [52, 60, 62]. Go4Health analyses interpreted the calls of these marginalized communities as consistent with UHC anchored in the right to health, and with their advocacy in the wider policy discourse [10, 22, 52, 64].

### Global health governance

Global health governance and global governance for health were an early preoccupation for Go4Health, shaped by the objectives of the research but also the overlapping membership with the Joint Action and Learning Initiative on National and Global Responsibilities for Health, with its advocacy for a Framework Convention for Global Health [26, 65]. The focus on governance and Go4Health’s anticipation of new governance frameworks reflected recognition of the progressive erosion of WHO’s leadership, the rise of new players, the fragmentation of global governance, and the precedent established with the emergence of new structures and networks around the release of the MDGs [26, 66, 67]. In health, the ambitious expansion of the SDG health agenda—particularly the inclusion of non-communicable diseases—called for new governance [68, 69], as would the demands generated by Go4Health’s commitment to the right to health [68].

The fragmentation of global health governance, the contested capacity of WHO, and competition for financing in the post-2015 were linked as threatening global health governance in the minds of Go4Health authors. WHO, acknowledged “as a normative agency endowed with unprecedented constitutional powers” [66] had recently been criticized for its slow and inadequate response to the Ebola epidemic in 2014 [20, 66, 70]. Van de Pas and Van Belle [70] linked this unsatisfactory response to the WHO’s financial vulnerability, causing its priority setting process to be overly influenced by influential governments and private funders. WHO was not alone in this, with increased reliance on private funding also experienced by other UN institutions [71]. Gostin et al. reiterated this concern: the lack of its own adequate budget, coupled with “a crowded and often chaotic global health architecture” [66], has limited WHO’s capacity to exercise its normative authority and fulfill its roles properly [67, 70]. Lack of funding, earmarked funding, conflicting demands from Member States, weak governance and excessive regionalization were identified as causes undermining the WHO’s global leadership [66]. Contributing to a shift in global health governance was the increasing use of ‘multi-bi’ development assistance, allowing bilateral donors (and others) to use their funding through multilateral channels to substantially reshape the agendas of civil society, multilateral partners, and domestic governments, at times to the advantage of their own foreign affairs interests [67].

But the human rights orientation of the Go4Health researchers was also reflected in their analysis of global health governance, and the issues they addressed: international solidarity and equity in response to shared global threats or persisting regional challenges [8, 43, 53, 72]; the formalization of global social contracts [8, 10, 50]; protection of the health rights of migrants and refugees [47]; and the protection of access to medicines for the disadvantaged [73, 74].

For Go4Health, committing to the right to health will necessarily have implications for meeting extra-territorial obligations—obligations largely discounted with the renewed emphasis on sovereign responsibility and control [43, 53]. In filling the funding gaps to deal with the health effects of climate change, a “fair share” would expect the global North to shoulder a significant component of the burden [8]. The potential influence of global social contracts was not to be dismissed [8, 10, 50], with the African Union’s 2012 Abuja Declaration *Roadmap on Shared Responsibility and Global Solidarity for AIDS, Tuberculosis and Malaria Response in Africa* providing a regionally driven example [50]. The collaboration evident in development of the SDGs should be extended to their implementation: the manipulation of health expenditure conditionalities in negotiating development assistance needs to give way to a model of “people-centred accountability” [10, 50, 57]. With conflict, globalization, economic and environmental conditions heightening international migration and displacement, Go4Health argued for a right to health approach for non-nationals, through extension of UHC to non-citizens within sovereign borders [47]. Civil society also plays a crucial independent role promoting the inclusion of marginalized peoples [46, 52], but the locus of responsibility under global health governance must remain the national government. Efforts to strengthen health systems and human rights protection needed engagement with governments, without which achieving UHC anchored in the right to health would be unrealistic [8, 10, 26, 43, 44, 47, 49, 53, 75]. And the currently accepted shared international responsibility for climate change must include shared responsibility for health, if we are to address the complexities of these interrelated policies effects on personal health and a healthy planet [72]. This shared international responsibility for health would be explored by Go4Health researchers in the imperatives for interdependence articulated in the SDGs.

### Integration of health across other SDGs

With health only one goal of the 17 SDGs, advocates could no longer assume the centrality for health granted in the MDGs, and health would only be a priority across the SDG agenda if integral to the achievement of the other SDGs [40, 76]. As such, Go4Health researchers advocated engagement across the entire SDG agenda, giving attention to the SDG goals of economic development, environment sustainability and social inclusion necessary to achieve the right to health. This rhetoric revisits a paradigm where health is seen as a pre-condition for, rather than merely the result of, sustainable social development. In this paradigm, “Ensure healthy lives and promote well-being for all at all ages” should focus on health and wellbeing rather than treatment of disease [40]. But given the path dependence implied in addressing the “unfinished business of the MDGs” [31, 68], the format of the nine SDG3 targets has reiterated the same disease targeted approach, further reinforced by the selection of indicators. Although the increased scope of the SDGs now arguably addressed most elements of the global disease burden [68], Go4Health was concerned that they did not adequately exploit the multi-sectoral potential of the SDGs. Only by engaging in the SDGs beyond health would the underlying determinants of health be addressed, optimizing the direct and indirect benefits for “ensuring health and well-being for all” that would be the product of the SDG’s sustainable environmental, social, and economic development [31, 69, 76].

Here there is an apparent tension: Go4Health international health rights advocates, having argued that realizing the right to health is both dependent on the structures of UHC, together with the interdependence expressed in the full scope of sustainable development [68], now express concern that the right to health may “drown” in a sea of competing SDGs [31]. Only the explicit inclusion of the right to health in the SDGs would have enabled that inter-sectoral dynamic, while ensuring the centrality of health [31, 40].

But despite being the fundamental position of Go4Health, this defining of the realization of the right to health as being both the implementation of UHC and the creation of a healthy environment [22], was not reflected in its publications. The implications of the impact of an unhealthy environment on health were only developed in a single commentary [72], and despite a richly multidisciplinary team, the journals that published Go4Health research were primarily in the public health domain. Go4Health, advocating for engagement across the diverse SDGs, effectively limited its dialogue to the global health community. But if Go4Health failed to significantly reach an audience beyond health, the inclusion of both human rights advocates and international health lawyers in the research had a conceptual and methodological impact on Go4Health research, that has broader potential in global health and development [44].

## Conclusions

Compared to the opacity of the development processes underlying the MDGs, the comprehensive consultation initiated by the UN to determine the global goals to succeed them has been intentionally transparent. That relative transparency, and the invitation to global participation in shaping the “World We Want”, has provided a platform on which contemporaneous policy research could be undertaken and the results fed into the developing discourse.

The analysis of the product of the Go4Health research confirms the potential of funding health systems and policy research to explore issues of global impact: Go4Health clearly influenced the global discourse, and in particular, contributed to diplomatic and policy responses and brought human rights to the debate. It contributed significantly to the discourse around its other themes—with high penetration in health related search engines. Based on the metric analysis, Go4Health publications were highly accessible in health related databases for each of the themes identified (Scopus, Web of Science, EMBASE, Global Health Library, PubMed).

As policy analysts, Go4Health authors claimed both research and advocacy as appropriate roles. Direct impact on the discourse is difficult to prove, though analysis of citing institutions between 2012 and 2016 points to Go4Health research being recognized by the major academic institutions. Go4Health contributed to the UN Thematic Consultation on Health [8], and policy influence is suggested by six citations in UN publications, ten citations by authors affiliated with WHO, and four by the Thai Ministry of Public Health, and the analysis of the right to health in the development of the SDGs cited in a UN resolution [42]. As part of the research agreement, European Commission policy advisors were regularly briefed on the findings of the research. Presentation at international conferences extended Go4Health’s scientific and diplomatic reach.

Evidently, the impact on the discourse did not entirely depend on the success of its positions: Go4Health was unsuccessful in securing UHC and “the realization of the right to health for everyone” as the overarching health goal. Its advocacy did not ensure overt reference to the right to health in the goals themselves. Despite strongly advocating the need for health to look outward and engage in the rich diversity of the other SDGs [21], there was limited penetration in the academic literature other than for health: for Worldwide Political Science Abstracts, even the transdisciplinary issues of the right to health had limited impact. The complementary contribution of sustainable social, economic and environmental development into “ensuring health and well-being for all” did not translate into a substantial research focus. And in a multidisciplinary team, bringing together academics and civil society, human rights lawyers and advocates with social scientists and public health researchers, tensions occasionally emerged over the balance of research and advocacy, between action and analysis [59].

Those tensions have been creative in terms of recognizing the synergies available to such a multidisciplinary team, researching collaboratively over a five-year period. Ooms and Hammonds [44] point to the epistemological differences between the normative analysis of international human rights lawyers and their civil society advocates and the empirical framing of public health researchers—particularly in mixed syllogisms that combine the normative of “what should be” with the descriptive of “what is”. This became particularly apparent in the exploration of the right to health in the SDGs and UHC in particular. The advocacy of UHC as a goal, “grounded in the right to health” [8, 10, 22]; the exploration of the extent that UHC embodies the right to health [38, 45]; the influence of human rights on the SDGs and global governance more broadly [38, 45, 47, 51, 53, 55, 68]—these analyses essentially hold the post-2015 development discourse to a normative right to health benchmark. In contrast, the empirical paradigm has dominated the research on multilateral agencies’ positioning on the right to health in the developing post-2015 discourse [39, 40]; and marginalized communities’ perceptions of the needs they want addressed under its global social contract [60–63]. But between these poles sit the kinds of synergies that Ooms and Hammonds [44] refer to, the intersection of the epistemological differences between right to health and public health in the strategies to address: global maternal mortality [43, 50]; tiered pricing for essential medicines [73, 74]; the underpinning of the global response to health security [26, 66, 70]; global governance for health [57, 67, 68]. It is the developing interplay of these two disparate framings over the life of the Go4Health project that speak to its essential contribution to the post-2015 discourse: that the collaboration, the integration of the normative with the empirical in shared explorations of its research themes, added these synergies to the post-2015 debate, and perceptibly shaped the discourse.

## Abbreviations

Go4Health: Goals and Governance for Health (Research Project)
MDG: Millennium Development Goal
Rio + 20: United Nations Conference on Sustainable Development
SDG: Sustainable Development Goal
UHC: Universal Health Coverage
UN: United Nations
WHO: World Health Organization

## References

[1] Ghaffar A, Gilson L, Tomson G, Viergever R, Røttingen J-A (2016). Where is the policy in health policy and systems research agenda?. Bull World Health Org.

[2] Adam T, Ahmad S, Bigdeli M, Ghaffar A, Røttingen J-A (2011). Trends in health policy and systems research over the past decade: still too little capacity in low-income countries. PLoS One.

[3] Mills A (2011). Health policy and systems research: defining the terrain; identifying the methods. Health Policy Plan.

[4] Walt G, Gilson L (1994). Reforming the health sector in developing countries: the central role of policy analysis. Health Policy Plan.

[5] Lehmann U, Gilson L (2012). Actor interfaces and practices of power in a community health worker programme: a south African study of unintended policy outcomes. Health Policy Plan.

[6] Buse K (2008). Addressing the theoretical, practical and ethical challenges inherent in prospective health policy analysis. Health Policy Plan.

[7] Sabatier PA, Weible CM, Sabatier PA (2007). The advocacy coalition framework: innovations and clarifications. Theories of the policy process.

[8] Go4Health (2012). The post-2015 international health agenda: universal health coverage and healthy environment, both anchored in the right to health.

[9] Go4Health. Goals and governance for health. http://www.go4health.eu/. Accessed 25 Jan 2018.

[10] Go4Health (2013). Realizing the right to health for everyone: the health goal for humanity.

[11] Go4Health. Publications. http://www.go4health.eu/publications. Accessed 25 Jan 2018.

[12] Liamputtong P (2013). Qualitative research methods. 4th edition.

[13] Green J, Thorogood N (2013). Qualitative methods for health research.

[14] QSR International. What is NVivo? http://www.qsrinternational.com/nvivo/what-is-nvivo. Accessed 25 Jan 2018.

[15] Worldwide Political Science Abstracts. http://www.proquest.com/products-services/polsci-set-c.html. Accessed 25 Jan 2018.

[16] Hicks D, Wouters P, Waltman L, De Rijcke S, Rafols I. The Leiden manifesto for research metrics. Nature 2015;520(7548):429. 10.1038/520429a 25903611

[17] ELSEVIER. SciVal. https://www.elsevier.com/solutions/scival. Accessed 25 Jan 2018.

[18] University of Leeds. Measuring the reach of your publications using SciVal: University of Leeds; 2017.

[19] United Nations. United Nations Official Document System*.* https://documents.un.org/prod/ods.nsf/home.xsp. Accessed 25 Jan 2018.

[20] Moon S, Sridhar D, Pate MA, Jha AK, Clinton C, Delaunay S (2015). Will Ebola change the game? Ten essential reforms before the next pandemic. The report of the Harvard-LSHTM independent panel on the global response to Ebola. Lancet.

[21] Hill PS, Buse K, Brolan CE, Ooms G (2014). How can health remain central post-2015 in a sustainable development paradigm?. Glob Health.

[22] Ooms G, Brolan C, Eggermont N, Eide A, Flores W, Forman L (2013). Universal health coverage anchored in the right to health. Bull World Health Org.

[23] WHO (2010). World health report 2010: health systems financing: the path to universal coverage.

[24] WHO (2012). Positioning health in the post-2015 development agenda: WHO discussion paper.

[25] WHO, World Bank (2015). Tracking universal health coverage: first global monitoring report.

[26] Brolan CE, Hill J, Hill PS (2013). Global governance for universal health coverage: could a framework convention on Global Health hold it together?. Glob Health Gov.

[27] Schmidt H, Gostin LO, Emanuel EJ (2015). Public health, universal health coverage, and sustainable development goals: can they coexist?. Lancet.

[28] United Nations (2013). The future we want: outcome document adopted at Rio+20.

[29] UN. Resolution adopted by the General Assembly on 12 December 2012: 67/81 Global health and foreign policy. http://www.un.org/en/ga/search/view_doc.asp?symbol=A/RES/67/81. Accessed 25 Jan 2018.

[30] WHO (2013). Sixty-sixth world health assembly: health in the post-2015 development agenda.

[31] Go4Health (2014). The health-related development goal in the post 2015 negotiations: the right to health – buoyed or drowning in sustainable development?.

[32] Hill PS (2015). The comeback of universal health coverage in the post-2015 debate?.

[33] Global Thematic Consultation on Health (2013). Health in the Post-2015 agenda.

[34] United Nations (2013). A new global partnership: eradicate poverty and transform economies through sustainable development.

[35] Sustainable Development Solutions Network (2013). An action agenda for sustainable development.

[36] Open Working Group (2013). Progress report of the open working Group of the General Assembly on sustainable development goals.

[37] United Nations (2015). Transforming our world: the 2030 agenda for sustainable development.

[38] Ooms G, Latif LA, Waris A, Brolan CE, Hammonds R, Friedman EA (2014). Is universal health coverage the practical expression of the right to health care?. BMC Int Health Hum R.

[39] Brolan CE, Hill PS, Ooms G (2015). “Everywhere but not specifically somewhere”: a qualitative study on why the right to health is not explicit in the post-2015 negotiations. BMC Int Health Hum R.

[40] Brolan CE, Hill PS (2016). Universal health Coverage’s evolving location in the post-2015 development agenda: key informant perspectives within multilateral and related agencies during the first phase of post-2015 negotiations. Health Pol Plan.

[41] Brolan CE, Te V, Floden N, Hill PS, Forman L (2017). Did the right to health get across the line? Examining the United Nations resolution on the sustainable development goals. *BMJ*. Glob Health.

[42] United Nations (2016). General assembly seventy-first session a/71/304: right of everyone to the enjoyment of the highest attainable standard of physical and mental health.

[43] Hammonds R, Ooms G (2014). The emergence of a global right to health norm–the unresolved case of universal access to quality emergency obstetric care. BMC Int Health Hum R.

[44] Ooms G, Hammonds R (2014). Right to health and global public health research: from tensions to synergy?. Tropical Med Int Health.

[45] Forman L, Ooms G, Chapman A, Friedman E, Waris A, Lamprea E (2013). What could a strengthened right to health bring to the post-2015 health development agenda?: interrogating the role of the minimum core concept in advancing essential global health needs. BMC Int Health Hum R.

[46] Meisterhans N (2016). Health for all: implementing the right to health in the Post-2015 agenda. Perspectives from the global south. *Social*. Medicine.

[47] Brolan CE, Dagron S, Forman L, Hammonds R, Latif LA, Waris A (2013). Health rights in the post-2015 development agenda: including non-nationals. Bull World Health Org.

[48] Ruano AL, Friedman EA, Hill PS (2014). Health, equity and the post-2015 agenda: raising the voices of marginalized communities. Int J Equity Health.

[49] Hussain S, Ruano AL, Rahman A, Rashid SF, Hill PS (2015). From knowing our needs to enacting change: findings from community consultations with indigenous communities in Bangladesh. Int J Equity Health.

[50] Ooms G, Mulumba M, Hammonds R, Laila AL, Waris A, Forman LA (2013). Global social contract to reduce maternal mortality: the human rights arguments and the case of Uganda. Reprod Health Matters.

[51] Forman L, Ooms G, Brolan CE (2015). Rights language in the sustainable development agenda: has right to health discourse and norms shaped health goals?. Int J Health Policy Manag.

[52] Friedman EA, Baba JT, Brolan CE, Chew A, Eggermont N, Gostin LO, Hasunira R, Hussain S, Latif L, Nantaba J, Nasreen H, Rahman A, Rashid SF, Sheridan S, Siddiqui F, Waris A, Hill P (2013). Community consultations on the post-2015 global health agenda: a demand for dignity, respect, participation and accountability.

[53] Ooms G, Hammonds R (2014). Global constitutionalism, responsibility to protect, and extra-territorial obligations to realize the right to health: time to overcome the double standard (once again). Int J Equity Health.

[54] Brolan CE, Hill PS (2014). Sexual and reproductive health and rights in the evolving post-2015 agenda: perspectives from key players from multilateral and related agencies in 2013. Reprod Health Matters.

[55] Sridhar D, McKee M, Ooms G, Beiersmann C, Friedman E, Gouda H (2015). Universal health coverage and the right to health from legal principle to Post-2015 indicators. Int J Health Serv.

[56] Rodney AM, Hill PS (2014). Achieving equity within universal health coverage: a narrative review of progress and resources for measuring success. Int J Equity Health.

[57] Ooms G, Sridhar D, Jahn A (2013). Global health governance after 2015. Lancet.

[58] Sridhar D, Brolan CE, Durrani S, Edge J, Gostin LO, Hill P (2013). Recent shifts in global governance: implications for the response to non-communicable diseases. PLoS Med.

[59] Brolan CE, Hussain S, Friedman EA, Ruano AL, Mulumba M, Rusike I (2014). Community participation in formulating the post-2015 health and development goal agenda: reflections of a multi-country research collaboration. Int J Equity Health.

[60] Baba JT, Brolan CE, Hill PS (2014). Aboriginal medical services cure more than illness: a qualitative study of how indigenous services address the health impacts of discrimination in Brisbane communities. Int J Equity Health.

[61] Mulumba M, Nantaba J, Brolan CE, Ruano AL, Brooker K, Hammonds R (2014). Perceptions and experiences of access to public healthcare by people with disabilities and older people in Uganda. Int J Equity Health.

[62] Ruano AL, Sánchez S, Jerez FJ, Flores W (2014). Making the post-MDG global health goals relevant for highly inequitable societies: findings from a consultation with marginalized populations in Guatemala. Int J Equity Health.

[63] Ibell C, Sheridan SA, Hill PS, Tasserei J, Maleb M-F, Rory J-J (2015). The individual, the government and the global community: sharing responsibility for health post-2015 in Vanuatu, a small island developing state. Int J Equity Health.

[64] Ooms G (2013). Health at a crossroad. UNA-UK. Global development goals: leaving no one behind.

[65] Hoffman SJ, Røttingen J-A (2013). Dark sides of the proposed framework convention on Global Health's many virtues: a systematic review and critical analysis. Health Hum Rights.

[66] Gostin LO, Sridhar D, Hougendobler D (2015). The normative authority of the World Health Organization. Pub Health.

[67] Sridhar D, Brolan CE, Durrani S, Edge J (2013). Governance and financing of global public health: the Post-2015 agenda. Brown J World Aff.

[68] Van de Pas R, Hill PS, Hammonds R, Ooms G, Forman L, Waris A, Brolan C, McKee M, Sridhar D (2017). Global Health governance in the sustainable development goals: is it grounded in the right to health?. Global Challenges.

[69] Hill PS. More is less? Health in the sustainable development goals. The Conversation. 2015;23 https://theconversation.com/more-is-less-health-in-the-sustainable-development-goals-47627. Accessed 30 Jan 2018

[70] Rvd P, Sv B (2015). Ebola, the epidemic that should never have happened. Glob Affairs.

[71] Meisterhans N (2014). An alternative model of governance?. Dev Cooperation Frankfurter Societäts-Medien.

[72] Ooms G, Brolan C, Jahn A, Hill P. Climate change and the shared responsibility for global health [letter]. Lancet Globl Health Blog. 2013; http://globalhealth.thelancet.com/2013/09/19/climate-change-and-shared-responsibility-global-health. Accessed 30 Jan 2018

[73] Ooms G, Forman L, Williams OD, Hill PS (2014). Could international compulsory licensing reconcile tiered pricing of pharmaceuticals with the right to health?. BMC Int Health Hum R.

[74] Williams OD, Ooms G, Hill PS (2015). Cautionary notes on a global tiered pricing framework for medicines. Am J Public Health.

[75] Waris A, Latif LA (2015). Towards establishing fiscal legitimacy through settled fiscal principles in global health financing. Health Care Anal.

[76] Brolan CE, Hill PS (2015). Countdown for health to the post-2015 UN sustainable development goals. Med J Aust.

